# Liddle syndrome due to a novel mutation in the γ subunit of the epithelial sodium channel (ENaC) in family from Russia: a case report

**DOI:** 10.1186/s12882-019-1579-4

**Published:** 2019-10-26

**Authors:** Anastasiya A. Kozina, Tatiana A. Trofimova, Elena G. Okuneva, Natalia V. Baryshnikova, Varvara A. Obuhova, Anna Yu. Krasnenko, Kirill Yu. Tsukanov, Olesya I. Klimchuk, Ekaterina I. Surkova, Peter A. Shatalov, Valery V. Ilinsky

**Affiliations:** 1 Institute of Biomedical Chemistry, Pogodinskaya street 10 building 8, 119121 Moscow, Russia; 2 Pirogov Russian National Research Medical University, Ostrovitianova street 1, 117997 Moscow, Russia; 3 Veltischev Research and Clinical Institute for Pediatrics of the Pirogov Russian National Research Medical University, Taldomskaya str 2, 125412 Moscow, Russia; 4 Genotek Ltd., Nastavnicheskii pereulok 17/1, 105120 Moscow, Russia; 5 Vavilov Institute of General Genetics, Gubkina street 3, 119333 Moscow, Russia

**Keywords:** Liddle syndrome, ENaC, *SCNN1G*, Pseudoaldosteronism, Hypertension

## Abstract

**Background:**

Liddle syndrome is a monogenic disease with autosomal dominant inheritance. Basic characteristics of this disease are hypertension, reduced concentration of aldosterone and renin activity, as well as increased excretion of potassium leading to low level of potassium in serum and metabolic alkalosis. The cause of Liddle syndrome is missense or frameshift mutations in *SCNN1A*, *SCNN1B*, or *SCNN1G* genes that encode epithelial sodium channel subunits.

**Case presentation:**

We describe a family with Liddle syndrome from Russia. 15-year-old proband has arterial hypertension, hypokalemia, hyporeninemia, metabolic alkalosis, but aldosterone level is within the normal range. At 12 years of age, arterial hypertension was noticed for the first time. We identified novel frameshift mutation c.1769delG (p.Gly590Alafs) in *SCNN1G,* which encodes the **γ** subunit of ENaC in vertebrates. The father and younger sister also harbor this heterozygous deletion. Treatment with amiloride of proband and his sister did not normalize the blood pressure, but normalized level of plasma renin activity.

**Conclusions:**

Our results expand the mutational spectrum of Liddle syndrome and provide further proof that the conserved PY motif is crucial to control of ENaC activity. Genetic analysis has implications for the management of hypertension, specific treatment with amiloride and counselling in families with Liddle syndrome.

## Background

Liddle syndrome (pseudoaldosteronism, OMIM 177200; 618,114; 618,126) is a genetically heterogeneous autosomal dominant disorder. The key clinical characteristics of this syndrome are early onset salt-sensitive hypertension with low level of K^+^, metabolic alkalosis, inhibition of renin activity and aldosterone secretion [1].

Mutations (missense or frameshift) in the genes of epithelial sodium channel (ENaC) subunits cause the Liddle syndrome. These channels belong to the ENaC/DEG (degenerin) family of proton-gated cation channels. ENaC mediates transport of Na^+^ through the apical membrane from lumen into the epithelial cell [2]. Since ENaC regulates Na^+^ ion balance in the extracellular fluid (ECF) and in the kidney, these channels are a significant part of ECF volume and blood pressure (BP) regulation [3].

ENaC channels are present in the apical portion of epithelial cells of distal nephron, distal colon, lung and ducts of exocrine glands. The channel consists of three subunits encoded by three genes: α (*SCNN1A*), β (*SCNN1B*), and γ (*SCNN1G*). All subunits have similar structure: cytoplasmic N-terminus, extracellular loop, two short hydrophobic segments (transmembrane domains 1 and 2) and cytoplasmic C-terminus. The N- and C-termini are turned to the cytosolic surface, whereas the extracellular loop is turned to the extracellular surface [4]. C-terminus of all ENaC subunits has a highly conserved sequence - PY (Proline Tyrosine) motif [5]. Gene knockout studies inactivating the ENaC subunits genes in mice demonstrated that all three subunits are significant for survival. Knockout mice without any ENaC subunit die due to respiratory insufficiency or kidney dysfunction [6, 7]. Similar symptoms were observed in humans with pseudohypoaldosteronism, type 1 (OMIM 264350). This disease develops if the patient has two mutant copies of one of the ENaC subunits genes and is allelic to Liddle syndrome [8].

Liddle syndrome is associated with germline mutations in an allele of *SCNN1A*, *SCNN1B* or *SCNN1G* genes [9]. These genes are located on different chromosomes: *SCNN1A* is on chromosome 12p13.31, *SCNN1B* and *SCNN1G* are on chromosome 16p12.2 [4]. The most mutations were found in *SCNN1B* and *SCNN1G* genes. Single mutation was reported in the gene *SCNN1A*: Salih et al. described a heterozygous missense mutation in the extracellular domain of the α-subunit of ENaC as the cause of Liddle syndrome [10]. Pathogenic variants in *SCNN1B* and *SCNN1G* remove or modify the intracellular Proline-Tyrosine motifs in ENaC [9].

Here, we describe a Russian family suffering from the Liddle syndrome due to frameshift mutation in the *SCNN1G* gene.

## Case presentation

We describe a Russian family with Liddle syndrome: proband, his mother, father and sister. Below is a detailed description of the clinical features of the proband and his sister. The basic biochemical characteristics of these patients are summarized in Table 1.

**Table 1:** Biochemical characteristics of proband and his sister carrying *SCNN1G* mutation before and after amiloride treatment. PAC – plasma aldosterone concentration, PRA – plasma renin activity

Patient	Sex	Age	BP, mm Hg average (maximum)	PAC, pg/mL	Normal range of PAC, pg/mL	PRA, ng/mL/h	Normal range of PRA, ng/mL/h	K+, mmol/L	Normal range of K+, mmol/L
Proband before amiloride treatment	M	15	149/95 (159/109)	88,968	10–160 pg/ml	0,1	1,2-2,4	3,4	3,7-5,12
Proband after amiloride treatment		16	140/90 (160/110)	15		2,13		4,8	
Sister of proband before amiloride treatment	F	14	120/80 (160/100)	9,7		1,01		4,0	
Sister of proband after amiloride treatment		15	115/69 (141/98)	10		2		4,7	

The proband is a 15-years-old male from Russian republic of Dagestan. He was born from the second pregnancy and second childbirth of healthy nonconsanguineous parents. His birth weight was 3500 g. The neonatal period was uneventful. He grew and developed according to his age.

During sport activities at 12 years of age, arterial hypertension was found for the first time. From 13.5 years, the patient has been complaining of headache and dyspnea during exercises. His BP was stable at 160/100 mmHg. Hypertrophy of the left ventricular myocardium and angiopathy of the retinal vessels were revealed. The plasma level of thyroid hormones, ACTH (adrenocorticotropic hormone), cortisol, dehydroepiandrosterone, aldosterone (23 pg/ml, normal range 10–160 pg/ml), adrenaline, noradrenaline, dopamine, and serotonin was normal. Urine level of metanephrines was normal. Serum level of renin activity was reduced to 0,1 ng/mL/h (normal range 1,2–2,4 ng/mL/h). Treatment with Lisinopril (inhibitor of angiotensin-converting enzyme, ACE) and Amlodipine (angioselective calcium channel blocker) was ineffective.

He was admitted to Veltischev Research and Clinical Institute for Pediatrics of the Pirogov Russian National Research Medical University (Moscow, Russia) at 14, 15 and 16 years.

On admission at 14 years he had high BP (160/120 mmHg), reduced level of potassium (3,4 mmol/L, normal range 3,7–5,12 mmol/L) and renin activity (0,5 ng/mL/h). Ultrasound showed no abnormality of kidney. Treatment with Amlodipine and Bisoprolol (beta-blocker) did not normalize hypertension.

During a year, the patient received Irbesartan, Amlodipine, Bisoprolol and Moxonidine. But arterial hypertension persisted.

On admission at 15 years his weight was 70 kg, height was 175 cm. Intellectual development corresponded to his age. Maximum BP was 159/109 mmHg. Heart rate while lying was 66 bpm, heart rate while standing was 76 bpm. The heart was not enlarged; heart sounds were clear and rhythmic. Biochemical analysis showed reduced level of potassium (3,4 mmol/L), reduced renin activity (0,1 ng/mL/h), elevated level of lactate dehydrogenase (LDH, 479 U/L, normal range 0–450 U/L) and creatine kinase (CK, 317 U/L, normal range 15–190 U/L). The acid-base balance is shifted towards metabolic alkalosis: pH 7,45, BE (Base Excess) + 7,4 mmol/L, HCO_3_ std. 31 mmol/L. Other biochemical parameters (levels of sodium, creatinine and others) as well as the aldosterone level (88,968 pg/ml), thyroid gland hormonal profile and cardiac indicators were within the normal range. Urine analysis was without any significant changes.

Echocardiography showed concentric left ventricular hypertrophy, tricuspid and mitral valve regurgitation. Global systolic and diastolic myocardial functions were not abnormal. Daily monitoring of BP showed stable systolic and diastolic arterial hypertension during night and daytime hours. Abdominal ultrasound revealed no significant changes.

Due to the presence of hypertension, hypokalemia, hyporeninemia, and metabolic alkalosis monogenic form of arterial hypertension was expected in patient. Differential diagnosis was conducted between two conditions: Liddle syndrome and syndrome of apparent mineralocorticoid excess.

The younger sister (14 years old, 46 kg, 162 cm) of proband had normal BP (120/80 mmHg) with a periodic increase to 160/100 mmHg. From 12 years, she has been complaining of headache and nausea in the morning. She had a reduced serum level of aldosterone (9,7 pg/mL), reduced renin activity (1,01 ng/mL/h) and normal level of potassium (4,0 mmol/L). The level of sodium (141 mmol/L) and cortisol (12,5 mcg/dL) were within the normal range. There were no signs of metabolic alkalosis in the sister’s blood (pH 7,4; BE − 3,4 mmol/L, HCO_3_ std. 22 mmol/L). Urine analysis was without any significant changes. Echocardiography showed symmetrical left ventricular hypertrophy.

The father of proband (47 years) also had arterial hypertension (BP 170/120 mmHg with periodic increase to 210/140 mmHg) since the age of 18. Ultrasound showed kidney cysts. It is impossible to describe detailed clinical symptoms of the father due to his refusal to undergo an examination and treatment.

Genomic DNA was extracted from peripheral blood samples of proband, his mother, father and younger sister.

Sequencing of clinically relevant genes (clinical exome) was done by Genotek Ltd. The Genotek’s Ethics Committee (08/2018) approved all studies. Parents gave written informed consent to research and publication of photos, clinical and sequencing data of themselves, proband and his sister.

NEBNext Ultra DNA Library Prep Kit for Illumina (New England Biolabs, MA, USA) was used as reagent for preparation of DNA libraries. Target enrichment was performed with SureSelect XT2 (Agilent Technologies, CA, USA) system. Next samples were sequenced on Illumina HiSeq 2500 (paired-end 100 bp reads). To trim 3′-nucleotides, we used Cutadapt [11]. Reads were aligned to GRCh37.p13 with BWA MEM [12]. Analyzing reads with FASTQS for quality control was performed [13]. We called short variants using GATK HaplotypeCaller [14] in concordance with GATK Best Practices DNA-seq [15, 16]. Variant effects were assessed by snpEff [17]. To estimate pathogenicity, information was taken from the dbNSFP [18], Clinvar [19], OMIM [20] and HGMD [21] databases. Limitations of in silico prediction tools - SIFT and PolyPhen-2 - did not allow us to use these instruments for this type of mutations. Mutant allele frequencies were extracted from 1000Genomes [22], ExAC [23] and Genotek databases. Pathogenicity was evaluated in accordance with international recommendations: ACMG (American College of Medical Genetics and Genomics), CAP (College of American Pathologists), AMP (Association for Molecular Pathology) [24].

All of the exons of the SCNN1A, SCNN1B and SCNN1G genes were sequenced. An additional table file shows other genes and exons that have been sequenced [see Additional file 1].

*SCNN1G* pathogenic variant was identified in proband and his relatives using exome sequencing (clinical exome) and then was verified by Sanger sequencing.

After exome sequencing proband and his sister were commenced on amiloride (with hydrochlorothiazide) and low-salt diet.

A dose of 5 mg of amiloride /50 mg of hydrochlorothiazide caused severe side effects (weakness, fatigue) in proband. The dose of the drug was reduced to 2,5 mg of amiloride /25 mg of hydrochlorothiazide. After 6-months therapy, BP of proband did not normalize with a maximum increase to 160/110. Also, elevated level of homocysteine was revealed (20,5 μmol/L, normal range 5–12 μmol/L). Other biochemical parameters as well as level of aldosterone and renin activity were within the normal range. However, ultrasound revealed diffuse changes in the renal parenchyma.

A dose of 5 mg of amiloride /50 mg of hydrochlorothiazide caused an increase in creatine levels up to 85 μmol/L in sister of proband. The dose of the drug was reduced to 2,5 mg of amiloride /25 mg of hydrochlorothiazide. After 6-months therapy, BP of the sister tended to normalize with a maximum increase to 141/98. Other biochemical parameters as well as level of aldosterone and renin activity were within the normal range. However, ultrasound revealed an increase in volume and thickening of the renal parenchyma.

## Discussion and conclusions

**Fig. 1:**
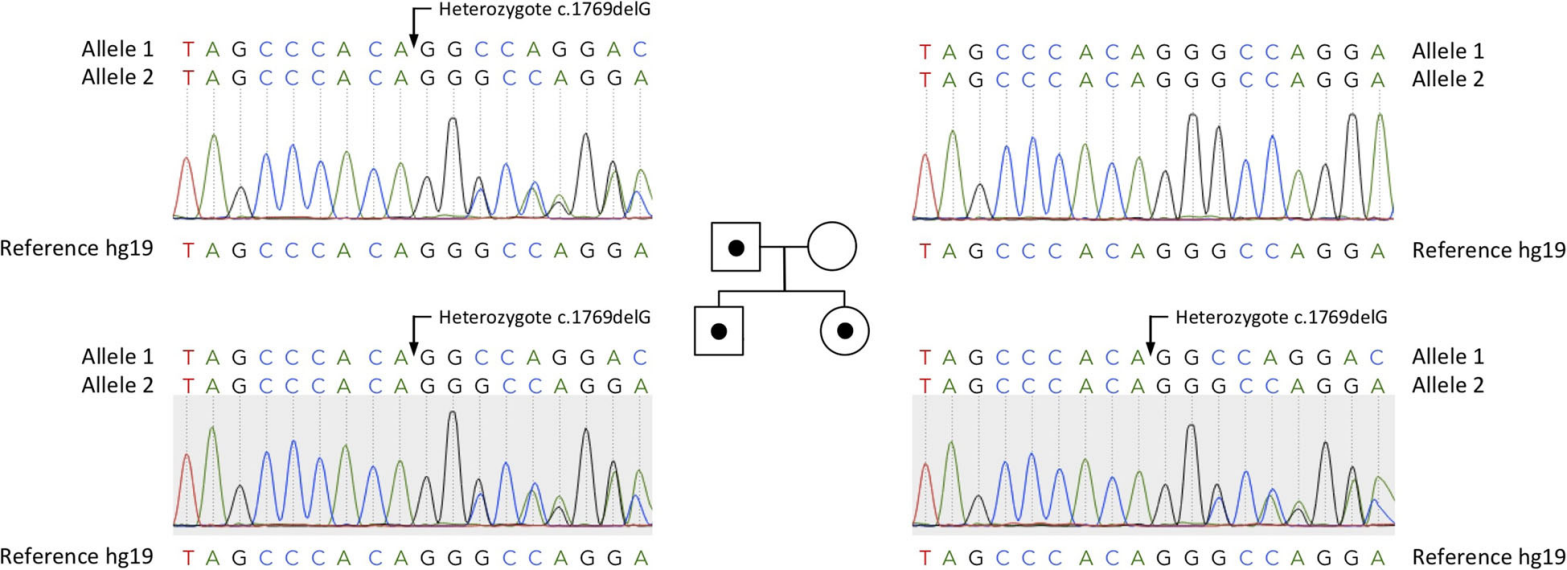
Pedigree of a family with Liddle syndrome. *Squares* and *circles* represent males and females, respectively. Individuals with the p.Gly590fs mutation are shown as *filled symbols*. Individuals lacking the mutation are shown as *open symbols*

We revealed novel heterozygous mutation c.1769delG (p.Gly590Alafs) of the *SCNN1G* (NM_001039.3) by NGS analysis and confirmed by Sanger sequencing. We identified this mutation in samples of proband, his father and sister. There was no mutation of the *SCNN1G* in mother (Fig. 1).

We also found that proband, his mother and sister have heterozygous missense mutation in *DSG2* gene: c.458A > G, p.Asn153Ser (NM_001943.3). According to the ACMG recommendation, this mutation is considered to be likely pathogenic. Mutation in this gene are regarded to cause arrhythmogenic (right ventricular) dysplasia (OMIM 610193). No other pathogenic variants and SNVs were found.

**Fig. 2:**
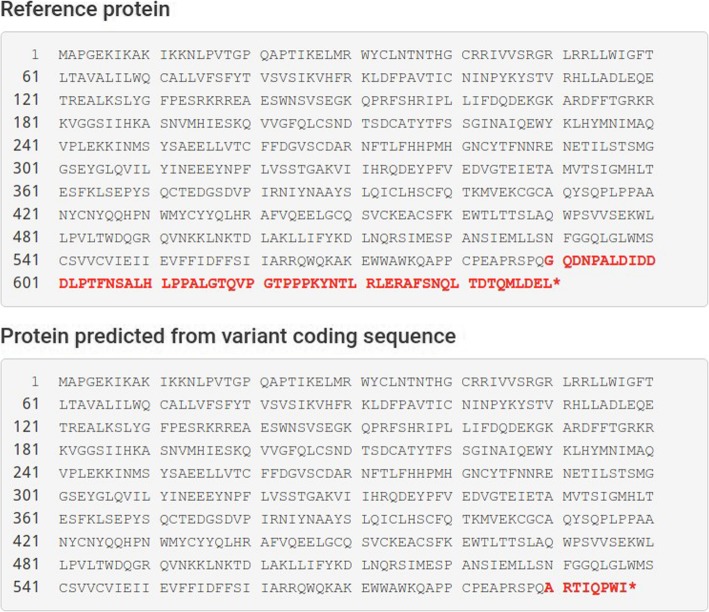
Prediction of changes in the γ subunit of ENaC channel due to c.1769delG mutation of *SCNN1G*

The c.1769delG mutation is deletion in exon 13 that shifted a reading frame. Mutalyzer [25] predicts that this variant leads to the removal of amino acids from 590 to 649 and replacement them with a short sequence of 8 amino acids (Fig. 2). This sequence ends with stop codon. According to the ACMG recommendation, c.1769delG mutation is considered to be likely pathogenic. This mutation has never been reported in the literature and databases (including ExaC and 1000 Genomes). Also, variant c.1769delG was not detected in 2000 patients of Genotek database.

Heterozygous mutations in the *SCNN1G* can lead to Liddle syndrome. To date, 6 different mutations of *SCNN1G* have been reported in 8 families from Finland, China and Japan [26–32] (Table 2).

**Table 2:** Comparison of described mutations in *SCNN1G* gene. The asterisk marks mutation described in this case report

Mutation	Clinical symptoms and source
c.1589A > G, p.Asn530Ser (N530S)	Finnish family: affected mother and son. Proband - 25-year-old man with hypertension, hypokalemia, low plasma renin activity and low serum aldosterone levels. Responded to amiloride or triamterene treatment [26].
c.1699C > T, p.Gln567Ter (Q567X)	Chinese family: 3 affected members [27].
c.1711G > T, p.Glu571Ter (E571X)	Chinese family: 5 affected members with hypertension, hypokalemia, low plasma renin activity and low serum aldosterone levels. Responded to amiloride treatment [28, 29].
c.1718G > A, p.Trp574Ter (W574X)	Japanese family: 4 affected members. Proband - 17-year-old woman with hypertension, hypokalemia, low plasma renin activity and low serum aldosterone levels. Responded to triamterene treatment and low-sodium diet [30].
c. 1724G > A, p.Trp576Ter (W576X)	Japanese family: de novo mutation Proband: 43-year-old woman with hypertension, metabolic alkalosis, hypokalemia, low plasma renin activity and low serum aldosterone levels. Responded to triamterene treatment [31].
c.1749_1753del, p.Glu583Aspfs (E583Dfs)	Chinese male patient with hypertension, metabolic alkalosis, hypokalemia, low plasma renin activity and normal serum aldosterone levels. Responded to triamterene treatment and low-sodium diet [32].
*c.1769delG, p.Gly590Alafs (G590A)	Russian family: 3 affected members Proband: 15-years-old boy with hypertension, hypokalemia, metabolic alkalosis, low plasma renin activity and normal serum aldosterone level Partially responded to amiloride treatment

Most of described mutations remove a proline rich segment (Proline-Tyrosine motif, PPPxY) in the carboxyl cytoplasmic tail of γ subunit (^624^PPPKY^628^). This segment is engaged in negative regulation of the channel and its overactivation [5]. PPPxY, serves as a region for connecting of Nedd4 (Neural precursor cell expressed developmentally down-regulated protein 4). This enzyme is ubiquitin-protein ligase and is involved in the internalization and the proteasomal degradation of the ENaC [33]. The removal of PPPxY inhibits the internalization and degradation of the channel using the ubiquitination-proteasomal pathway. Also, its leads to the accumulation of ENaC in the distal nephron apical membrane, which in turn increases sodium reabsorption [4].

The result of mutation p.Gly590Alafs is shortening of the C-terminus of the γ ENaC with absence of the PY motif. Termination occurs further along the amino acid sequence than in all described cases (Table 2). This allows us to decrease the limit for critical shortening of the γ subunit for Liddle syndrome. Hansson et al. reported p.Trp574Ter mutation in Japanese family with Liddle syndrome [30]. According to the Genome Browser [34] region between 574 and 590 amino acids is non-conservative. The missense mutations that change this region is unlikely to have a large impact on the protein function. Mutations leading to the premature stopping of protein synthesis with removal of PY motif cause a change channel function. Discovered by us mutation provides farther proof that the removal of conserved PY motif is crucial to function of ENaC subunits.

Using DNA sequencing analysis, we diagnosed the proband and his family. The permeability of cell membranes for sodium is significantly increased in patients with Liddle syndrome. Kidneys of these patients are in condition as if they were consuming and retaining excessive quantities of salt, and a low-salt diet is a significant part of therapy [35]. For the treatment of this syndrome, potassium-sparing diuretics, such as amiloride and triamterene, are needed. Amiloride and triamterene works by precisely blocking the ENaC. Therapy of Liddle syndrome with amiloride or triamterene reduces BP as well as corrects hypokalemia and alkalosis. Amiloride was prescribed to proband and his sister. Father refused treatment. Treatment with amiloride did not result in normalization of BP, but plasma renin activity has reached a normal level.

Classic phenotype of Liddle syndrome is characterized by severe hypertension and hypokalemia, but this disease can be clinically heterogeneous. Patients may have high BP without other symptoms. Genetic testing coupled with hormonal studies can help in the early detection of monogenic arterial hypertension. In our case, identification of *SCNN1G* mutation allowed to start therapy of the younger sister before development of resistant hypertension and pathological changes in the heart. Her BP after treatment with amiloride tends to normalize.

There are several forms of monogenic hypertension: Liddle syndrome, glucocorticoid-remediable aldosteronism, Gordon syndrome, apparent mineralocorticoid excess, congenital adrenal hyperplasia. Almost all these forms are characterized by damage to electrolyte transport in the distal nephron or the synthesis or activity of mineralocorticoid hormones [36].

Definition of the molecular basis of Liddle syndrome is helpful for early diagnosis, understanding the pathophysiology of the disease and selection of personalized therapy. The difficulty of identifying monogenic forms of hypertension increases the frequency of misdiagnosis. Misdiagnosis and incorrect treatment may cause early-onset stroke, terminal stage of renal failure, myocardial infarction and sudden death.

In summary, we have described a family suffering from the Liddle syndrome caused by a novel frameshift mutation (c.1769delG) in the gene encoding the γ subunit of the epithelial sodium channel.

## Supplementary information

**Additional file 1.** Genes and exons that have been sequenced in all patients. The file is a list of genes that were sequenced during the analysis, with indication of the number of sequenced exons for each gene.

## Data Availability

We did not use new software, databases, or applications/tools in the article. The datasets used and/or analyzed during the current study are available from the corresponding author on reasonable request.

## Abbreviations

ACE: Angiotensin-converting enzyme
ACMG: American College of Medical Genetics and Genomics
ACTH: Adrenocorticotropic hormone
AMP: Association for Molecular Pathology
CAP: College of American Pathologists
CPK: Creatine phosphokinase
ENaC: Epithelial sodium channel
LDH: Lactate dehydrogenase
